# Above- and Belowground Plant Functional Composition Show Similar Changes During Temperate Forest Swamp Succession

**DOI:** 10.3389/fpls.2021.658883

**Published:** 2021-06-28

**Authors:** Yu-Kun Hu, Xu Pan, Xu-Yan Liu, Zhi-Xi Fu, Man-Yin Zhang

**Affiliations:** ^1^Beijing Key Laboratory of Wetland Services and Restoration, Institute of Wetland Research, Chinese Academy of Forestry, Beijing, China; ^2^Hengshuihu National Wetland Ecosystem Research Station, Hengshui, China; ^3^Key Laboratory of Ecosystem Network Observation and Modeling, Institute of Geographic Sciences and Natural Resources Research, Chinese Academy of Sciences, Beijing, China; ^4^College of Life Sciences, Sichuan Normal University, Chengdu, China

**Keywords:** **** above- and belowground linkages, chronosequence, community-weighted mean, fine roots, functional diversity, functional strategies, functional traits

## Abstract

Plant functional composition, defined by both community-weighted mean (CWM) traits and functional diversity, can provide insights into plant ecological strategies and community assembly. However, our understanding of plant functional composition during succession is largely based on aboveground traits. Here we investigated community-level traits and functional diversity for six pairs of analogous leaf and fine root traits of understory plants in a temperate forest swamp during succession with a decrease in soil pH and nutrient availability. CWMs of traits related to resource acquisition (including specific leaf area, specific root length, leaf N, leaf P, root N, and root P) decreased with succession, whereas those related to resource conservation (leaf dry matter content, root dry matter content, leaf tissue density, leaf C, and root C) increased along the forest swamp successional gradient. Multi-trait functional dispersion (FDis) of both leaf and fine root traits tended to decrease along the successional gradient, but functional richness and evenness were highest at the middle successional stage. Moreover, FDis of individual plant traits except N showed the same pattern as multi-trait FDis. Soil pH and nutrient availability were the main drivers of successional changes in both CWM traits and FDis. The changes of community-level traits along succession indicated a shift from acquisitive to conservative strategy of understory plants during forest swamp succession. Similar trends in leaf and fine root functional diversity along succession may indicate above- and belowground functional diversity are coordinated during the processes of plant community assembly. These findings of linkages between above- and belowground plant functional composition have important implications for plant community dynamics and assembly rules.

## Introduction

Knowing how plant functional traits and their diversity vary under different environmental conditions would not only provides insight into species coexistence, but also aids our understanding of vegetation shifts in response to environmental changes (Mason et al., 2012; Bruelheide et al., 2018). During succession, the physical environment always shows predictable changes (Odum, 1969; Wang, 2002; Walker et al., 2010). Significant changes in environmental factors filter out plant species which cannot tolerate the modified conditions; consequently, the surviving plants have certain trait values (Díaz et al., 1998). Thus, community-level plant traits always showed clear trends along successional gradients (Holdaway et al., 2011). On the other hand, variation in physical environments as well as biotic interactions within communities may account for changes in functional diversity during succession (Zemunik et al., 2015; Craven et al., 2018). For example, Lohbeck et al. (2012) found that incidence-based functional diversity of multiple leaf and wood traits increased logarithmically with succession of tropical secondary forests. Plant functional diversity of leaf and regenerative traits was also observed to increase along an arable-to-grassland chronosequence (Purschke et al., 2013). In contrast, Bhaskar et al. (2014) found that how plant functional diversity of seasonally dry tropical forests changed after management activities depended on the traits studied (i.e. specific leaf area, leaf N, and wood density). These studies have made significant contributions to our understanding of the diversity of aboveground functional traits along successional or environmental gradients. However, community-level traits and functional diversity for fine root traits along environmental gradients is much less well understood (but see Holdaway et al., 2011; Fry et al., 2018; Caplan et al., 2019), which greatly hinders investigation of community assembly operating on belowground plant communities.

Belowground plant functional traits, especially fine root traits, define plant water, and nutrient acquisition strategies, which strongly influence species performance and dynamics (Laliberté, 2017; Caplan et al., 2019). From a whole-plant perspective, it would be insightful to investigate responses of functional traits and diversity to environmental changes through consideration of both above- and belowground traits (Kleyer and Minden, 2015). However, our understanding of functional diversity of fine roots lags that of leaves, mainly because fine roots are more difficult to collect and measure (Pérez-Harguindeguy et al., 2013). A number of studies have reported strong coordination between leaf and root traits (Freschet et al., 2010; Liu et al., 2010; Valverde-Barrantes et al., 2017; Hu et al., 2019a) where patterns of means and diversity of functional root traits were thought to be similar to those of leaf traits. However, some studies have reported instances where leaf and root traits were weakly correlated (Geng et al., 2014; Marañón et al., 2020), such that root traits were considered more than just analogs of leaf traits (Bergmann et al., 2017; De Long et al., 2019). Thus, there is a need to further examine whether functional diversity of root traits follows the same patterns as that of leaf traits along environmental gradients.

Ecosystems along a chronosequence provide an ideal natural setup for studying temporal changes in plant functional diversity which are driven by environmental conditions and/or biological processes (Walker et al., 2010). Such chronosequences occur in forest swamps, which, however, have been largely overlooked from a functional trait perspective. Primary succession of temperate forest swamps is different from that of other ecosystems and the major drivers of successional changes are the overgrowth of moss and decrease in soil pH, which lower the soil nutrient availability (van der Valk, 2006; Rydin and Jeglum, 2013; Hu et al., 2019a). Furthermore, forest swamps are located in many parts of the boreal and temperate zones, and are vulnerable to environmental changes (Erwin, 2009). Studying the plant functional diversity of forest swamps during succession would help with understanding and predicting how vegetation will change under future environmental conditions.

In this study, we aimed to investigate how above- and belowground community-level traits and functional diversity of understory plant communities change during forest swamp primary succession. As the succession of temperate forest swamps was mainly characterized by the obvious changes in understory plants, here we only focused on the understory community. Forest swamps tend to shift from nutrient-rich to nutrient-poor conditions as succession progresses (Hu et al., 2019a), and plant functional strategies have been reported to be closely related to soil nutrients (Ordoñez et al., 2009; Zemunik et al., 2015). Thus, we first hypothesized that abundance-weighted resource-acquisitive plant functional traits would decrease with succession, while resource-conservative plant traits would increase regardless of whether they are leaf or root traits. Soil nutrients, environmental heterogeneity and species richness have been shown to be the main drivers of plant functional diversity (Mayfield et al., 2010; Lohbeck et al., 2012; Stark et al., 2017). Then, we hypothesized that leaf and fine root functional diversity should decrease similarly along the successional gradient due to the decreases in soil pH, soil nutrient availability and species richness. To test these hypotheses, we measured six pairs of analogous leaf and fine root traits related to carbon, nutrient and water economics in forest swamps at three different successional stages. Community-weighted means (CWMs) and three functional diversity indices were calculated and compared for leaf and fine root traits along the forest swamp successional gradient.

## Materials and Methods

### Study Region

The study site was located in the Lingfeng National Nature Reserve (52°15′–52°31′N, 122°41′–123°26′E) in the Greater Hinggan Mountains, NE China (Supplementary Figure 1). Part of the cold temperate zones, the region has a mean annual temperature of about −5°C and a mean annual precipitation of ca. 500 mm. The main soil types in this area include gelic cambosols, permagelic gleyosols, and orthic spodosols (Editorial Committee of Soil Geography in China, 2014). Within Lingfeng National Nature Reserve, as across the cold-temperate region, coniferous forests constitute the dominant vegetation in the terrestrial ecosystems. Forest swamps comprise the second largest vegetation type in the Greater Hinggan Mountains (Editorial Committee of Wetland Vegetation in China, 1999). The Emuer River flows across the Lingfeng National Nature Reserve, forming large areas of forest swamps in the riparian zone (Editorial Committee of Wetland Vegetation in China, 1999). We applied a space-for-time substitution approach and selected three forest swamps from across the riparian zone to represent a successional gradient (early, middle, and late stages) (Supplementary Figure 1). Although the accurate ages for the successional stages of forest swamps were not available, we used a rough estimation based on previous studies of spore and pollen records which was performed not far from our study region (<50 km) (Xia, 1996). The ages of chosen forest swamps ranged from the present day (early stage) to 1300 years old (late stage). The main drivers of forest swamp succession are the expansion of *Sphagnum*, decreases in soil pH and nutrient availability and changes in water supply (Rydin and Jeglum, 2013). The early successional stage (present day) was characterized by minerotrophic soils with a high soil pH and water-table level and dominant species such as *Larix gmelini*, *Betula platyphylla*, and *Carex schmidtii* (Hu et al., 2019a). The middle successional stage (hundreds of years) had intermediate soil nutrient availability and pH with a moderate water table level and an abundance of *L. gmelini*, *Vaccinium* spp., and mosses. The forest swamps representing the late successional stage (ca. 1300 years) had the lowest nutrient availability, soil pH and water-table level, and common species including *L. gmelini*, *Ledum palustre*, *Vaccinium vitis-idaea*, and *Sphagnum* (Editorial Committee of Wetland Vegetation in China, 1999; Hu et al., 2019a).

### Field Survey and Sampling

We performed all the field investigation and sampling during the peak growing season, between July 1 and 15, 2017. The three forest swamps we chose to represent succession across the riparian zone were located close to one another (<5 km apart). As described in section “Study Region,” there was a significant decrease in soil pH and nutrient availability along the successional gradient (Supplementary Figure 2; Rydin and Jeglum, 2013; Hu et al., 2019a). To determine the species composition, we randomly located five replicated plots (10 × 10 m) within each type of forest swamp (Supplementary Figure 1). Within each plot, we then recorded the percentage cover of the understory shrubs and herbs using two 2 × 2 m quadrats (corners) and five 1 × 1 m quadrats (center and corners), respectively. Based on percentage cover, we collected samples of the dominant and subordinate understory plants at each successional stage. Notably, species composition was determined at quadrat level and averaged into plot-level data, and the sampling and trait measurement was performed for species within each successional stage. In total, we sampled 19 vascular plant species (11, early; 5, middle; and 8, late), which together constituted more than 95% of the total cover per successional stage (Hu et al., 2019a). Both species identity and abundance of understory plants changed during forest swamp succession (see Supplementary Table 1 in Hu et al., 2019b for a species list).

For each species within each successional stage, three batches (replicates) of leaves were sampled; each batch was randomly collected from 5 to 10 adult individuals. Only leaves that were green, mature and without evidence of animal herbivory were collected. In addition, three batches of intact roots of each species were carefully collected at 0–20 cm soil depth. We randomly sampled roots from 3 to 15 adult individuals of each species at each successional stage. The roots of each species were excavated and identified based on the aboveground parts of plants. All sampled plant material was stored in sealed plastic bags, kept cool and measured for traits within 8 h of collection.

### Soil Variables

Five soil samples (0–20 cm) were randomly collected from each plot after clearing the litter layer on the soil surface. Approximately 10 g of each fresh soil sample was oven dried at 105°C for 6 h to determine soil water content (%). The other soil samples were air dried at room temperature (ca. 20°C) to constant weight and then analyzed for soil pH, soil total C, N, and P contents. Once dry, the soil samples were passed through a 2 mm sieve. To determine soil pH, 5 g of soil from each sample was shaken with 12.5 mL demineralized water in a glass beaker and left to stand for 30 min, after which pH was measured using a pH Meter (PB-10; Sartorius, Germany). Before measurement of soil C, N, and P contents, the soil samples were ground and passed through a 0.15 mm sieve. Soil total C and total N contents (mg g^–1^) were measured using an elemental analyzer (vario MICRO cube; Elementar, Germany), whereas soil total P content (mg g^–1^) was determined using inductively coupled plasma-optical emission spectrometry (ICP-OES Prodigy 7; Teledyne Leeman Labs, United States). Soil C:N ratio was calculated as the ratio of soil total C content to soil total N content.

### Leaf and Fine Root Traits

For each species at each of the three successional stages, we measured six pairs of analogous leaf and fine root traits related to acquisition and conservation of resources (i.e., carbon, nutrient, and water). A subsample of each batch of leaves and fine roots were used to measure the traits. The fresh weight of each subsample varied due to the size of leaves and fine roots of different species, i.e., 0.2–3 g for leaves and 0.5–2 g for roots. First, we carefully cut the fine roots (diameter <2 mm) off each fresh root subsample and weighed them. We then scanned the fresh leaf and fine root samples using a Cannon scanner (resolution: 400 dpi) and measured total leaf area and fine root length from the scanned images using ImageJ^1^ and WinRHIZO (PRO V.2004a; Regent Instruments, Canada), respectively. We also calculated root diameter (mm) using WinRHIZO. The fresh weights of the leaf and fine root samples were recorded, following which they were oven dried at 65°C for 72 h to determined their respective dry weights. Specific leaf area (SLA, mm^2^ mg^–1^) was calculated as the ratio of total leaf area to oven-dry weight, while specific root length (SRL, m g^–1^) was calculated as root length per unit of oven-dry weight. Leaf dry matter content (LDMC, mg g^–1^) and fine root dry matter content (RDMC, mg g^–1^) were estimated using the ratio of leaf and root oven-dry weight to fresh weight, respectively. Then, leaf (LTD, g cm^–3^) and fine root tissue density (RTD, g cm^–3^) were respectively calculated as leaf and root dry mass divided by the volume of the tissues. Leaf volume was calculated as the product of leaf area and thickness, and root volume was estimated as the product of root diameter and length. Lastly, the concentrations of C, N, and P (mg g^–1^) in the leaves and fine roots were determined using the same methods as for the measurement of soil C, N, and P contents.

### Community-Level Traits and Functional Diversity

To quantify community-level traits, we used the CWMs of trait values. For each of the five plots at each successional stage, the CWM for each trait was calculated as $\sum_{i=1}^{\text{n}}\left({\text{p}}_{\text{i}}\times {\text{trait}}_{\text{i}}\right)$, where p_i_ is the relative abundance of species i and trait_i_, the trait value of species i (Garnier et al., 2004). To quantify the functional diversity of understory plant communities, we first calculated functional dispersion (FDis) for each plot. We chose this index not only because it is a functional diversity index which can integrate both single and multiple traits, but also because it can include the relative abundance of species within a community (Villéger et al., 2008; Mouchet et al., 2010). The FDis index describes the average distance in multidimensional trait space of individual species to the centroid of all species (Laliberté and Legendre, 2010). Higher FDis values show higher variability in plant functional traits across species within communities after taking into account species abundance. We calculated abundance-weighted FDis in each plot for all leaf or fine root traits together, and separately for each individual trait. Because different plant traits have different units, trait values were standardized to zero mean and unit variance before calculating functional diversity. To better explore the successional changes in functional composition, we also calculated another two components of functional diversity, i.e., functional richness (FRic, the volume of trait space occupied by species within the community) and evenness (FEve, the regularity of the distribution of species abundance in the trait space) (Villéger et al., 2008; Mouchet et al., 2010). These two indices provided complementary information for functional diversity. Both CWMs and functional diversity were calculated using the *dbFD* function of the “*FD*” R package (Laliberté et al., 2014).

### Statistical Analysis

#### Changes in Community-Weighted Mean Traits of Understory Plants During Succession

To investigate the influences of succession on CWM traits of forest swamps, we carried out one-way analysis of variance (ANOVA) and Tukey’s multiple comparison tests for CWM trait values among successional stages. We then performed a principal component analysis (PCA) for CWM traits across the three forest swamp successional stages to investigate how community resource strategies shifted along the successional gradient. To test whether successional changes in CWMs were driven by soil variables, we did linear regression analyses to determine the relationships between soil factors and the first axis of PCA for CWM traits.

#### Changes in Functional Diversity of Understory Plants During Succession

Using two-way ANOVA, we examined the influences of successional stage and organ type on FDis for multiple traits and for individual traits. These analyses not only allowed us to investigate how plant FDis varied during succession, but also helped to test whether the successional changes in functional diversity depended on plant organ type. We then explored the changes in FRic, FEve, species richness, and soil factors among successional stages using one-way ANOVA and multiple comparison tests. In addition, we calculated the coefficient of variation (CV) for soil factors to represent soil heterogeneity (Stark et al., 2017) and then tested the effects of species diversity, soil factors, and soil heterogeneity on functional diversity by performing simple linear regressions to investigate the relationships between these variables. To further disentangle the direct and indirect effects of these biotic and abiotic factors, we carried out structural equation modeling (SEM) separately for leaf and for fine root functional diversity. Based on linear regression results and previous studies, we constructed an initial model that included soil pH, soil N, CV of soil N, and species richness as variables. The SEM process was conducted using the R package “*piecewiseSEM*” (Lefcheck, 2016). All data analyses were carried out using R version 3.4.3 (R Core Team, 2017).

## Results

### Changes in Community-Weighted Mean Traits of Understory Plants During Forest Swamp Succession

**FIGURE 1:**
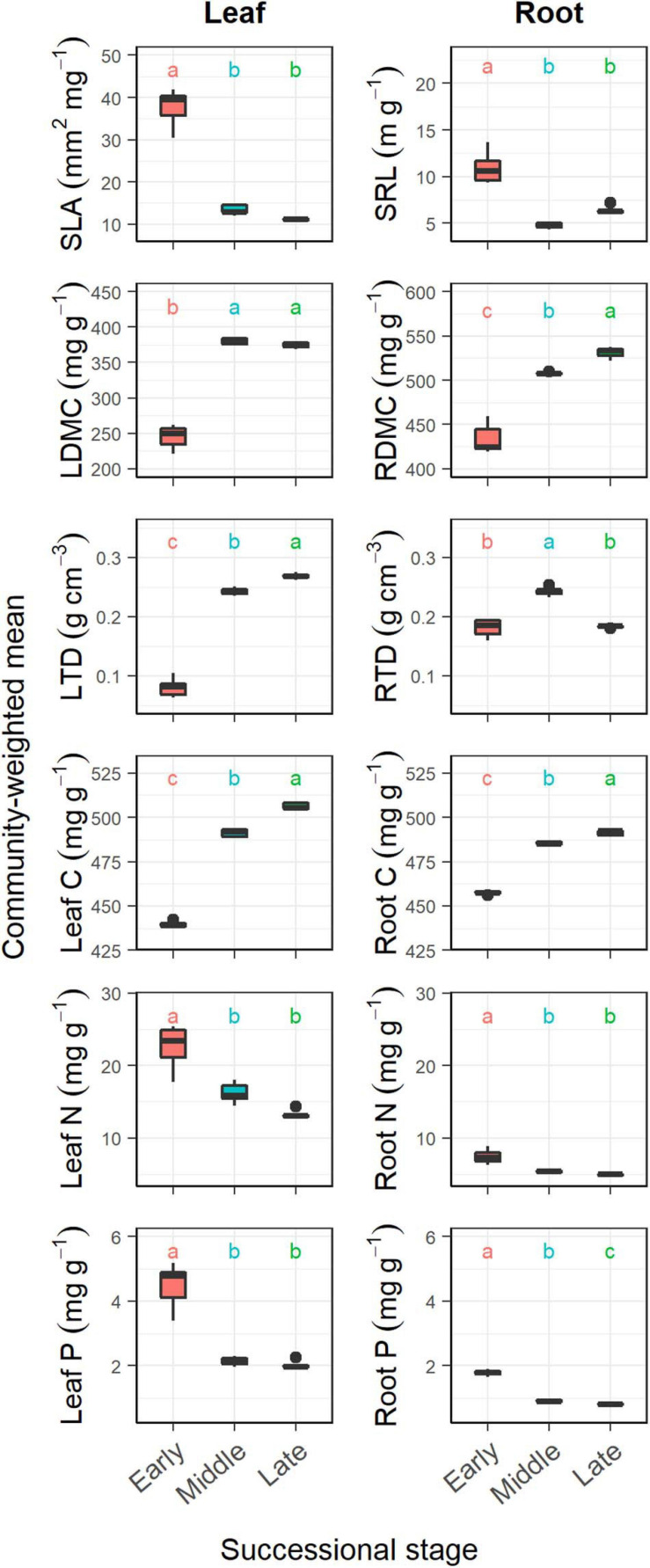
Changes in community-weighted means (CWMs) of plant traits during forest swamp succession. Boxplots denote interquartile range (box) and median (center line) of the values, and whiskers indicate the 95th and 5th percentiles. SLA, specific leaf area; SRL, specific root length; LDMC, leaf dry matter content; RDMC, root dry matter content; LTD, leaf tissue density; RTD, root tissue density. Different letters (a, b, and c) indicate significant differences in CWMs between successional stages (*p* < 0.05).

**FIGURE 2:**
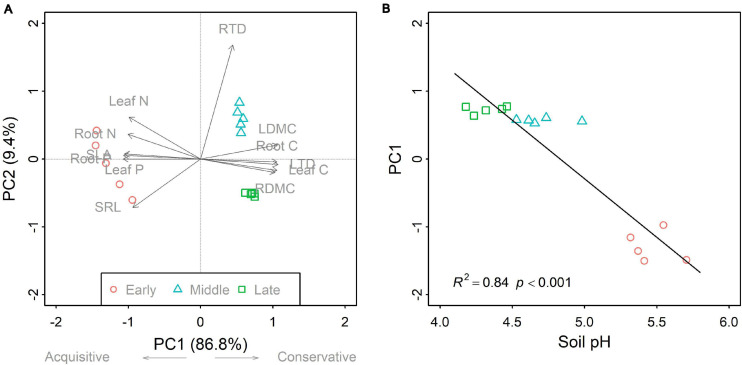
Principal component analysis for community-weighted mean (CWM) traits **(A)**, and the linear relationship between the first axis (PC1) and soil pH **(B)** across the three forest swamp successional stages. For **(A)**, PC1 explained 86.8% of the total variation, whereas the second axis (PC2) accounted for 9.4%. The PC1 was related to the plant economics spectrum, a continuum between acquisitive and conservative strategies. The linear relationship between soil pH and PC1 is evident in **(B)**. Full names for the abbreviations can be seen in Figure 1.

Overall, successional stage had significant effects on CWMs of all leaf and fine root traits (*p* < 0.05; Table 1). However, we observed contrasting changes in traits related to different resource strategies along the successional gradient (Figure 1). Traits related to resource acquisition, including SLA, leaf N, leaf P, SRL, root N, and root P, tended to decrease as succession progressed, although the CWMs of the middle and late successional stages were not significantly different (*p* > 0.05; Figures 1, 2). Being related to resource conservation, LDMC, leaf tissue density, leaf C, RDMC, and root C tended to increase along the successional gradient, although LDMC was not significantly different between the middle and late stages (*p* > 0.05; Figures 1, 2). In contrast, root tissue density increased significantly from early to middle stages but decreased from middle to late stages (Figure 1). These leaf and root traits concentrated mainly on axis 1 of the PCA, which accounted for 86.8% of the total variation (Figure 2). The PC1 of traits decreased significantly with increasing soil pH (*R*^2^ = 0.84, *p* < 0.001; Figure 2). In addition, there were similar changes in leaf and fine root traits related to the same resource strategies along the successional gradient (Figures 1, 2).

**TABLE 1:** Effects of successional stage and plant organ on community-weighted means (CWMs) and functional diversity (FDis) of plant traits in forest swamps.

**Factor**	**Successional stage**			**Plant organ**			**Successional stage × plant organ**		
	***df***	***F***	***p***	***df***	***F***	***p***	***df***	***F***	***p***
CWM*									
DMC	2	93.70	**<0.001**	1	506.14	**<0.001**	2	15.41	**<0.001**
TD	2	145.26	**<0.001**	1	297.42	**<0.001**	2	18.73	**<0.001**
C	2	429.90	**<0.001**	1	279.25	**<0.001**	2	34.22	**<0.001**
N	2	4.42	**0.024**	1	195.81	**<0.001**	2	1.00	0.383
P	2	25.61	**<0.001**	1	151.93	**<0.001**	2	5.38	**0.013**
***FDis***									
Multiple traits	2	22.07	**<0.001**	1	6.51	**<0.018**	2	1.24	0.309
SLA/SRL	2	15.45	**<0.001**	1	1.08	0.310	2	2.94	0.074
DMC	2	20.72	**<0.001**	1	0.02	0.880	2	0.27	0.763
TD	2	2.22	0.132	1	5.81	**0.025**	2	1.87	0.178
C	2	12.02	**<0.001**	1	5.64	**0.027**	2	6.32	**0.007**
N	2	31.02	**<0.001**	1	5.85	**0.024**	2	0.32	0.726
P	2	11.35	**<0.001**	1	34.10	**<0.001**	2	6.36	**0.007**

*No analysis for CWM of SLA and SRL due to the different units for these two traits. SLA, specific leaf area; SRL, specific root length; DMC, dry matter content; TD, tissue density. Significant effects (*p* < 0.05) are marked in bold.

### Changes in Functional Diversity of Understory Plants During Forest Swamp Succession

**FIGURE 3:**
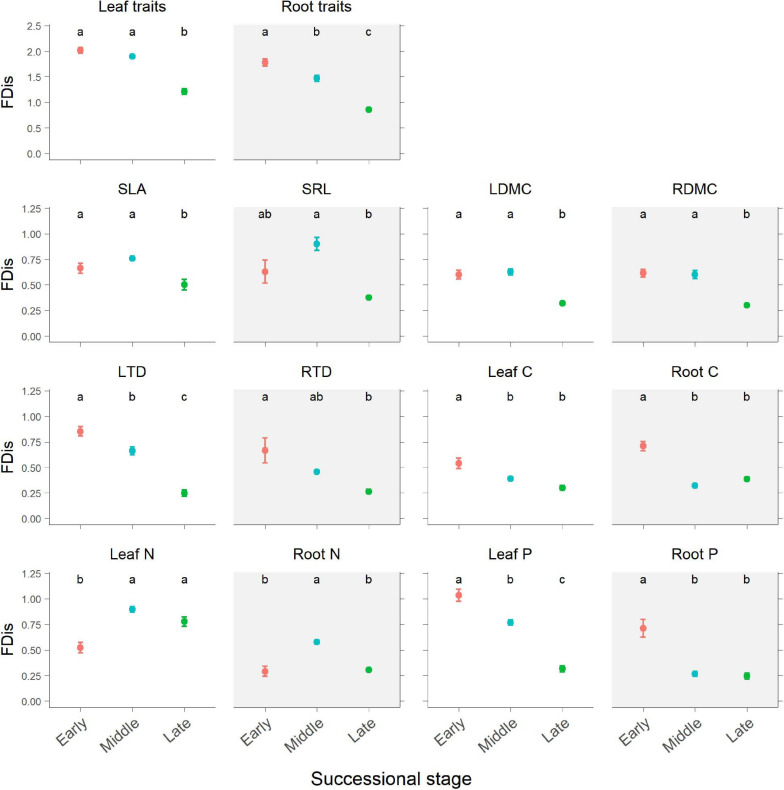
Changes in plant functional dispersion (FDis) of multiple and individual traits during forest swamp succession. Mean FDis values and standard errors are given for each successional stage. White background, leaf traits; gray background, root traits. Different letters (a, b, and c) indicate significant differences in functional dispersion between successional stages (*p* < 0.05). Full names for other abbreviations can be seen in Figure 1.

According to the results of ANOVA, successional stage had significant influences on FDis of both leaf and fine root traits (*p* < 0.05; Table 1 and Figure 3). There was no significant interaction between successional stage and plant organ on FDis of multiple traits as well as individual traits except for C and P (*p* > 0.05; Table 1). For multiple traits, both leaf and root FDis tended to decrease along the forest swamp successional gradient, although leaf FDis of the early and middle stages were not significantly different from each other (*p* > 0.05; Figure 3). As for individual traits, FDis of all leaf and root traits, except for N, decreased similarly along the successional gradient (Figure 3). For both leaf and root N, plant communities at the middle successional stage had the highest FDis (Figure 3).

In contrast to FDis, both FRic and FEve of plant traits were highest for plant communities at the middle successional stage (Supplementary Figure 3). However, both FRic and FEve of leaf and root traits showed similar changes during forest swamp succession, which are consistent with the result of FDis (Supplementary Figure 3).

### Soil Factors, Soil Heterogeneity, Species Richness, and Their Relationships With Functional Diversity of Understory Plants

**FIGURE 4:**
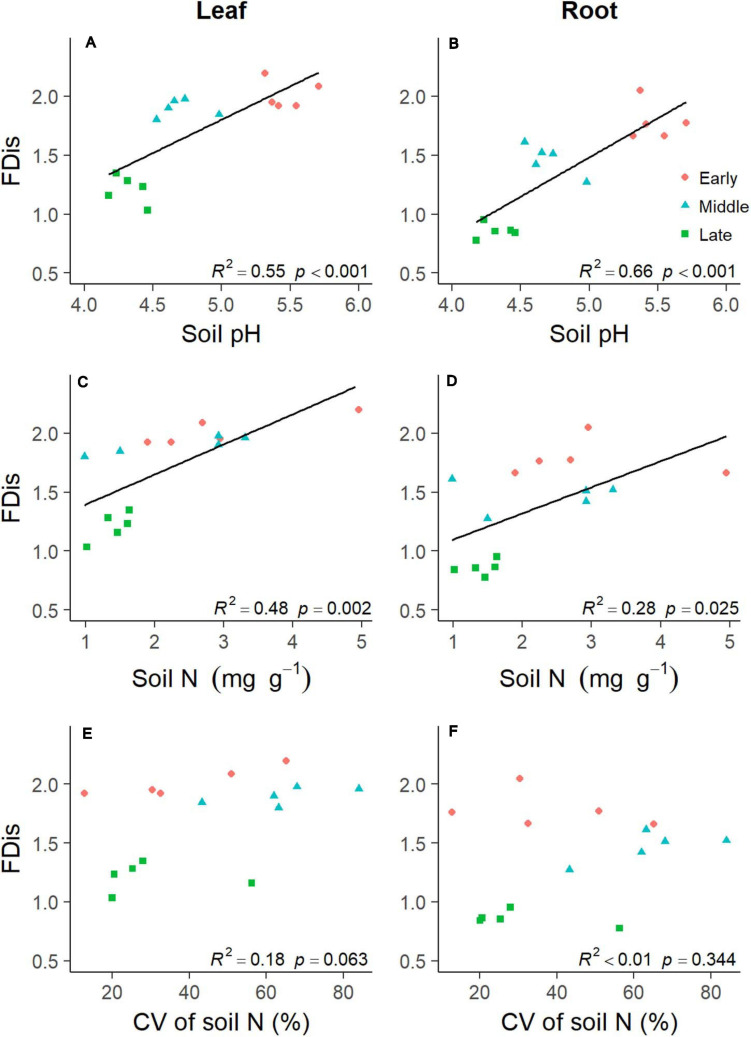
Relationships between plant functional dispersion (FDis) and soil properties [including soil pH **(A,B)**, soil N **(C,D)**, and coefficient of variation (CV) of soil N **(E,F)**] during forest swamp succession. Regression lines were added only for the significant relationships (*p* < 0.05).

**FIGURE 5:**
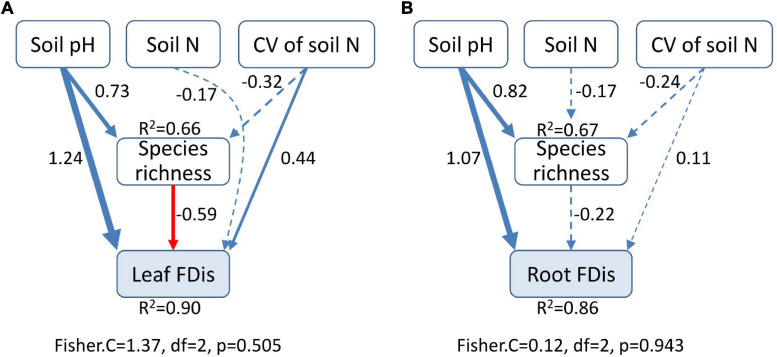
Structural equation modeling for the effects of soil pH, soil N, heterogeneity of soil N, and species richness on leaf **(A)** and root **(B)** functional dispersion (FDis). Heterogeneity of soil N was represented by coefficient of variation (CV). The width of lines is proportional to the standardized regression coefficients. Blue solid lines indicate significant and positive effects, while red solid lines represent significant and negative effects. Dashed lines denote no significant relationship. Results of goodness-of-fit tests are given (*p* > 0.05 indicates a good fit).

Soil properties changed during forest swamp succession (Supplementary Figure 2). Soil pH and soil N content showed significant positive relationships with FDis of both leaf and fine root traits (*p* < 0.05; Supplementary Table 1 and Figure 4). Even after controlling for other variables, soil pH was the best predictor of leaf and fine root FDis (Figure 5). The CV of soil factors varied along the successional gradient (Supplementary Figure 4), but the CV of soil N was not significantly correlated with FDis of leaf and root traits (*p* > 0.05; Supplementary Table 1 and Figure 4). On the other hand, plant species richness of forest swamps was significantly influenced by successional stage (*F*_2,12_ = 15.61, *p* < 0.05). Species richness of the early successional stage was significantly higher than that of the other two stages (*p* < 0.05; Supplementary Figure 2). The contribution of species richness to leaf and root FDis in forest swamps was weak both before and after controlling for the effects of other variables (Supplementary Table 1 and Figure 5).

## Discussion

### Plant Functional Traits and Resource Strategies During Succession

We found that CWM dry matter content, tissue density and C increased, and SLA, SRL, leaf, and root N and P decreased as the succession progressed in forest swamps. It has been largely known that dry matter content, tissue density and C involve resource conservation and physical defense and resistance to disturbances (Elger and Willby, 2003; Pérez-Harguindeguy et al., 2013), and that SLA, SRL, leaf, and root N and P are closely related to resource acquisition (Wright et al., 2004; Reich, 2014). Thus, those results support our first hypothesis, i.e., community-level resource-conservative traits tend to increase, and resource-acquisitive traits decrease with succession. These findings are consistent with the results of previous studies on primary succession in temperate rain forests (Holdaway et al., 2011), old-field succession (Shipley et al., 2006), and secondary succession in deciduous forests (Wilfahrt et al., 2014) and subtropical wet forests (Muscarella et al., 2016). All these studies concluded that plant communities at later successional stages tended to be more resource-conservative as light or nutrient availability decreased. In this study, the directional shifts in CWM plant traits with succession reflect the adaptation of plants to the decrease in soil pH and nutrient availability along the successional gradient.

Moreover, CWMs of analogous leaf and fine root traits showed similar trends along the successional gradient. This result is consistent with our expectation since leaf traits have been reported to be strongly coordinated with fine root traits at both community (Figure 2) and species level (Valverde-Barrantes et al., 2017; Hu et al., 2019a). It showed that there were similar plant strategy responses reflected in both the leaf and fine root traits. Notably, compared with species-level traits (Hu et al., 2019a), community-level plant traits showed more clear and predictable changes as succession progressed. This indicates the importance of integrating species relative abundance into studying and predicting plant community functional structure changes.

Small changes in community-level functional traits occurred between the middle and late successional stages, although these traits tended to show predictable shifts across all stages. There are two possible reasons for this result. One may be that these two later stages had a more similar species composition and denser shrub layer than the early stage (Hu et al., 2019a). Thus, forest swamps from the two later successional stages had more similar CWM traits. The other one may be related to the context or ecosystem differences. Different ecosystems could have different modes of succession. For example, Lohbeck et al. (2013) found contrasting successional changes in functional composition of dry and wet tropical forest, and Aiba et al. (2016) observed context-dependent changes in the functional composition of tree communities after land-use change along successional gradients. The factors including ecosystem type may increase the difficulty of predicting changes in plant functional composition during succession (Chang and Turner, 2019). Thus, future studies should focus more on examining plant functional traits across different ecosystems, in different contexts, and along environmental gradients, to improve the predictability of plant trait shifts in response to environmental changes (Bruelheide et al., 2018) and the understanding of plant-soil interactions (Holdaway et al., 2011).

### Above- and Belowground Plant Functional Diversity During Succession

Partially consistent with our expectation, multi-trait FDis of forest swamps decreased with succession, and this pattern coincided with the patterns of functional diversity of most individual traits, except for leaf and root N. These results were in agreement with the findings of Mason et al. (2012), but they are inconsistent with the findings of the majority of previous studies which reported that plant functional diversity tended to increase with succession (Lohbeck et al., 2012; Purschke et al., 2013; Zemunik et al., 2015; Craven et al., 2018), or showed no clear patterns (Böhnke et al., 2014; Laine et al., 2018). In contrast to FDis, FRic, and FEve based on leaf and root traits increased from the early to middle successional stage, then decreased from middle to late stage, indicating the dependence of functional diversity patterns on metrics. In our study, during forest swamp succession, some fast-growing and nutrient-acquisitive plant species (e.g., *C. schmidtii*, *Ribes nigrum*, and *Rubus arcticus*), characteristic of the early stage, were replaced by slow-growing and nutrient-conservative species (e.g., *L. palustre* and *V. vitis*-*idaea*) by the late successional stage (Hu et al., 2019a). The apparent decline in functional diversity during succession may reflect a convergence of plant community traits on resource-use strategies by the late stage which had lower soil pH, water and nutrient availability (Supplementary Figure 2). This finding indicates the important role of environmental filtering in limiting functional diversity during forest swamp succession, which is also supported by the strong explanation of soil pH and nutrient availability for functional diversity (Figures 4, 5). Inconsistent with our second expectation, FDis of leaf or root N was highest at the middle successional stage. That is because the most dominant species (i.e. *Vaccinium uliginosum* and *V. vitis*-*idaea*) at the middle stage had more contrasting leaf N (21.41 vs 10.99 mg g^–1^) and root N (4.68 vs 6.21 mg g^–1^) than dominant species (*L. palustre* and *V. vitis-idaea*) at the late stage (leaf N, 14.68 vs 9.10 mg g^–1^; root N, 3.95 vs 5.07 mg g^–1^). Overall, plant functional diversity showed directional changes during forest swamp succession. This study, focusing on a less studied ecosystem (forest swamp) and a different driver of ecological succession (soil pH), provides new evidence for the constraint of environmental filtering on plant functional diversity.

Similar successional patterns in functional diversity of leaf and fine root traits reflect the coupling of above- and belowground plant functional diversity. This is consistent with the coordination between community-level leaf and fine root traits found in this study. Moreover, functional diversity of leaves and fine roots also showed similar responses to soil properties (Figures 4, 5). As soil pH and nutrient availability decreased during succession, leaf and fine root functional diversity tended to be similarly affected by these soil properties. Our results support the idea that functional diversity of root traits can be predicted by that of leaf traits just as in many case species root traits could be inferred from leaf traits based on the strong correlations between them (Freschet et al., 2010; Liu et al., 2010; Valverde-Barrantes et al., 2017). The findings of congruent patterns for leaf and root functional diversity are inconsistent with the few studies which investigated both above- and belowground plant traits (Mason et al., 2012; Zemunik et al., 2015; de la Riva et al., 2018). Mason et al. (2012) found that functional diversity of aboveground plant traits decreased along a long-term soil chronosequence, and Zemunik et al. (2015) observed functional diversity of belowground traits related to nutrient acquisition increase along the same chronosequence. These different successional patterns for leaf and root traits may be related to the different calculation of functional diversity in these two studies. Meanwhile, de la Riva et al. (2018) found incongruent patterns of leaf and root morphological trait diversity along an arid gradient in Mediterranean woody communities. The different results for economic traits from this study and morphological traits from de la Riva et al. (2018) clearly showed that the patterns of leaf and root functional diversity could also depend on the trait dimension. As root traits have been found to be multidimensional (Marañón et al., 2020), it would be important to examine the coordination of leaf and root functional diversity based on other suites of traits, e.g., regenerative traits.

While very few of previous studies have investigated the successional shifts in functional diversity of belowground traits, our results found that leaf and root functional diversity decreased similarly during forest swamp succession. This finding has important implications for plant community assembly and ecosystem functions. First, similar trends in leaf and fine root functional diversity across succession suggest that assembly processes may operate similarly for above- and belowground compartments, at least for the resource economic dimension. Second, our finding would also aid the understanding of successional changes in ecosystem functions, because functional diversity of economic traits has been shown to determine many important ecosystem functions, e.g., productivity, carbon fluxes, and storage (Zuo et al., 2016; De Long et al., 2019).

## Conclusion

From a functional trait perspective, we studied the successional changes in above- and belowground compartments in temperate forest swamps. We observed similar shifts in CWMs of analogous leaf and fine root traits of understory plants during succession. Consistent with our first expectation, community-level traits related to resource acquisition decreased as succession progressed, whereas means of traits related to resource conservation increased along the successional gradient. On the other hand, FDis of both leaf and fine root traits (except for N) tended to decrease across successional stages. The shifts in functional diversity of multiple or single traits were similar for leaves and fine roots, which is partially consistent with our second expectation. This may be related to the similar responses of leaf and fine root functional diversity to soil properties. The similar temporal changes in functional diversity of leaves and fine roots indicate that above- and belowground functional diversity may be similarly driven by plant community assembly processes during succession. These findings of coupling between above- and belowground plant functional composition provide insights into community dynamics and ecosystem function in response to environmental changes.

## Data Availability Statement

Original species-level data for leaf and fine root traits is available from Dryad https://doi.org/10.5061/dryad.6cn55fb (Hu et al., 2019b). Data for community-weighted means and functional diversity are included in the Supplementary Material.

## Author Contributions

Y-KH conceived the study and wrote the first draft. Y-KH and X-YL collected the data. Y-KH and XP analyzed the data and made the figures. Y-KH, XP, X-YL, Z-XF, and M-YZ contributed to reviewing and editing the manuscript. All authors contributed to the article and approved the submitted version.

## Conflict of Interest

The authors declare that the research was conducted in the absence of any commercial or financial relationships that could be construed as a potential conflict of interest.

## Footnotes

1 https://imagej.nih.gov/ij/
