# Supplementary material for: Above- and Belowground Plant Functional Composition Show Similar Changes During Temperate Forest Swamp Succession
Source: Front Plant Sci. 2021 Jun 28;12:658883. doi: 10.3389/fpls.2021.658883 (PMC8274568; doi:10.3389/fpls.2021.658883)
Supplement: Supplementary file 1 [file Data_Sheet_1.docx]

***Supplementary Material***

**1 Supplementary Figures and Tables**

**1.1 Supplementary Figures**


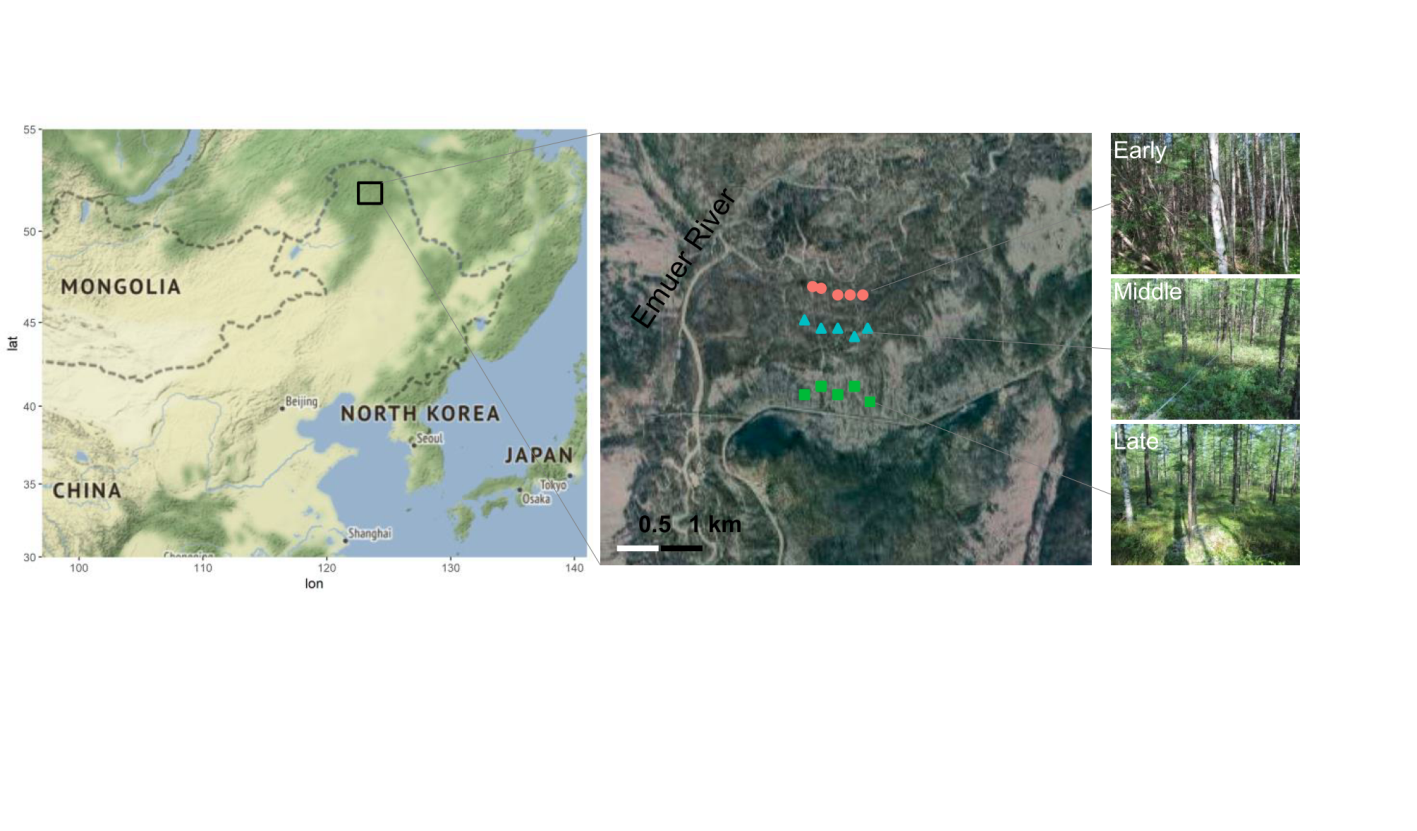


**Supplementary Figure S1.** Location of study region, plots, and pictures for forest swamps at different successional stages. Different symbols represent plots at different stages: ●, early; ▲, middle; ■, late.


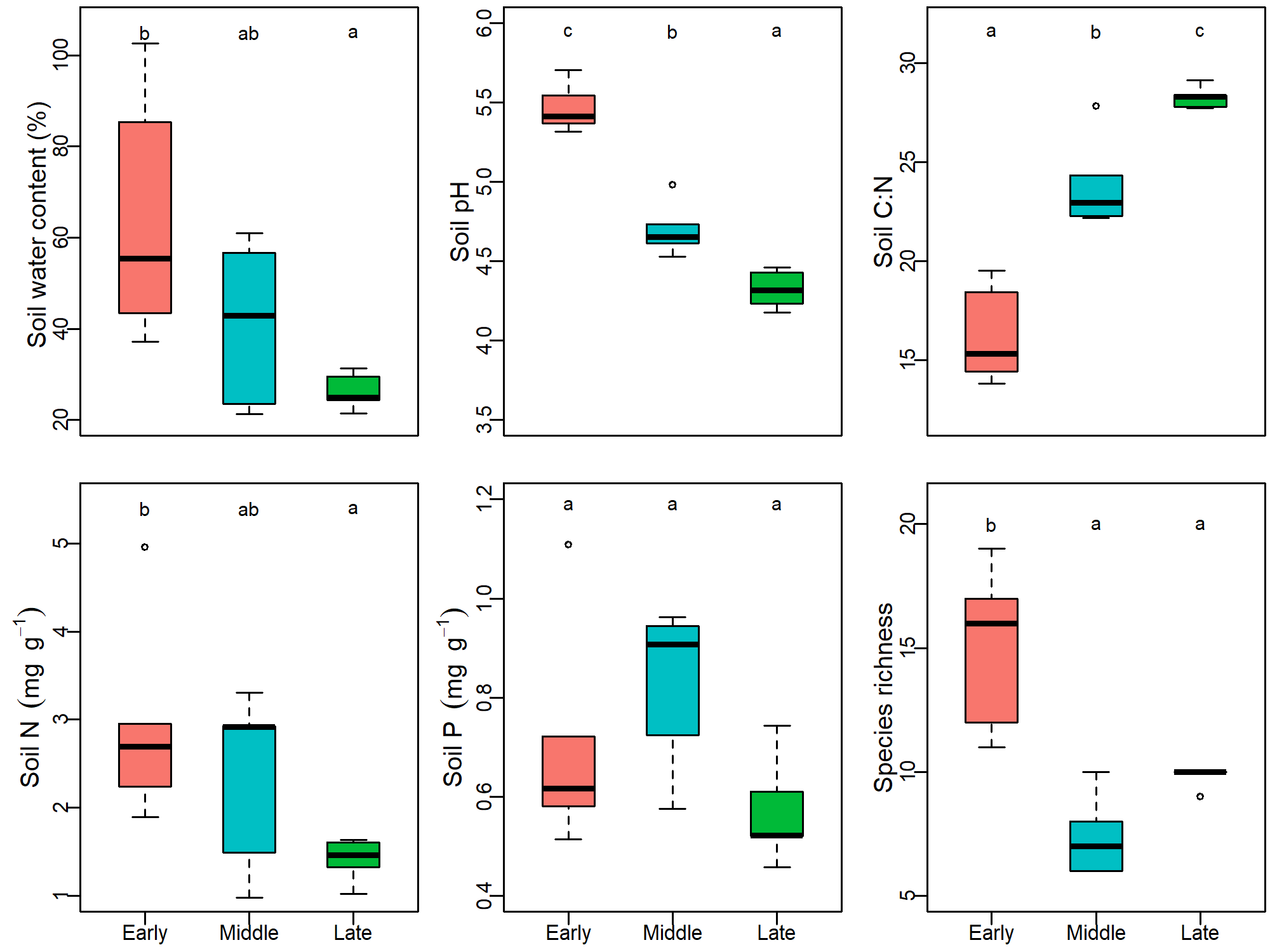


**Supplementary Figure S2.** Changes in soil properties during forest swamp succession. Different letters (a, b and c) indicate significant differences in soil properties between successional stages (*p* < 0.05).


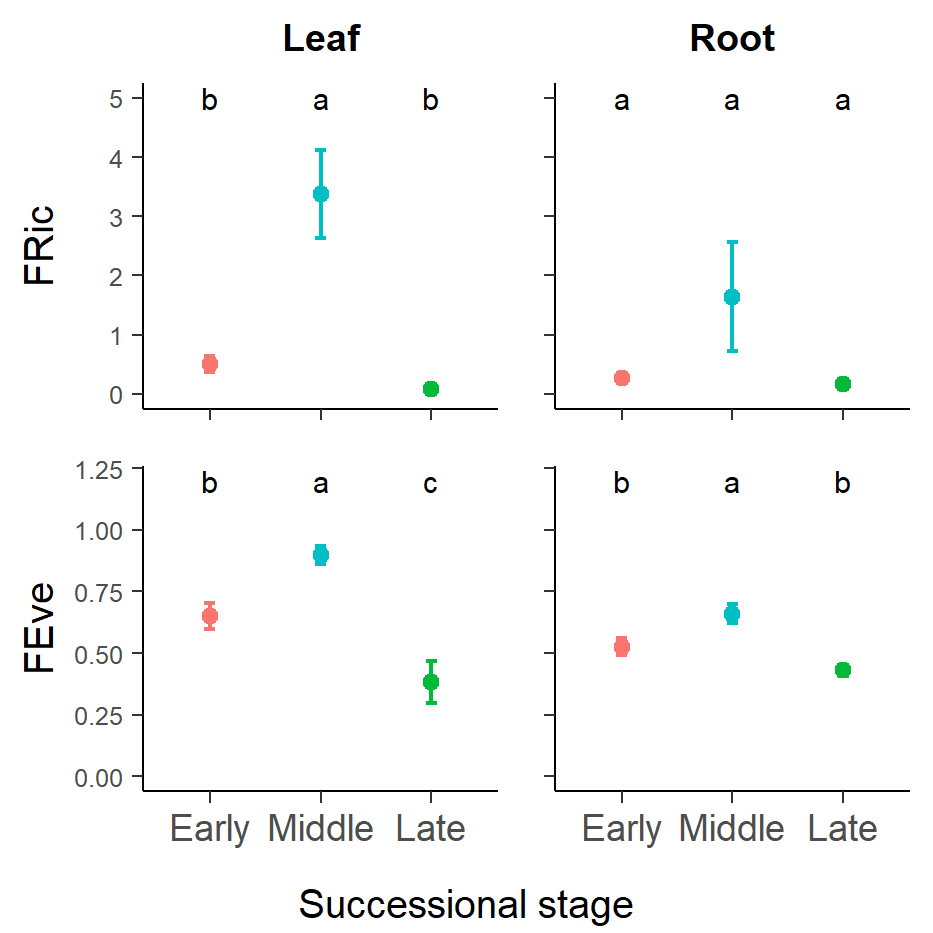


**Supplementary Figure S3.** Changes in functional richness (FRic) and evenness (FEve) of leaf and root traits during forest swamp succession. Mean FRic or FEve values and standard errors are given for each successional stage. Different letters (a, b and c) denote significant differences in FRic or FEve values between successional stages (*p* < 0.05).


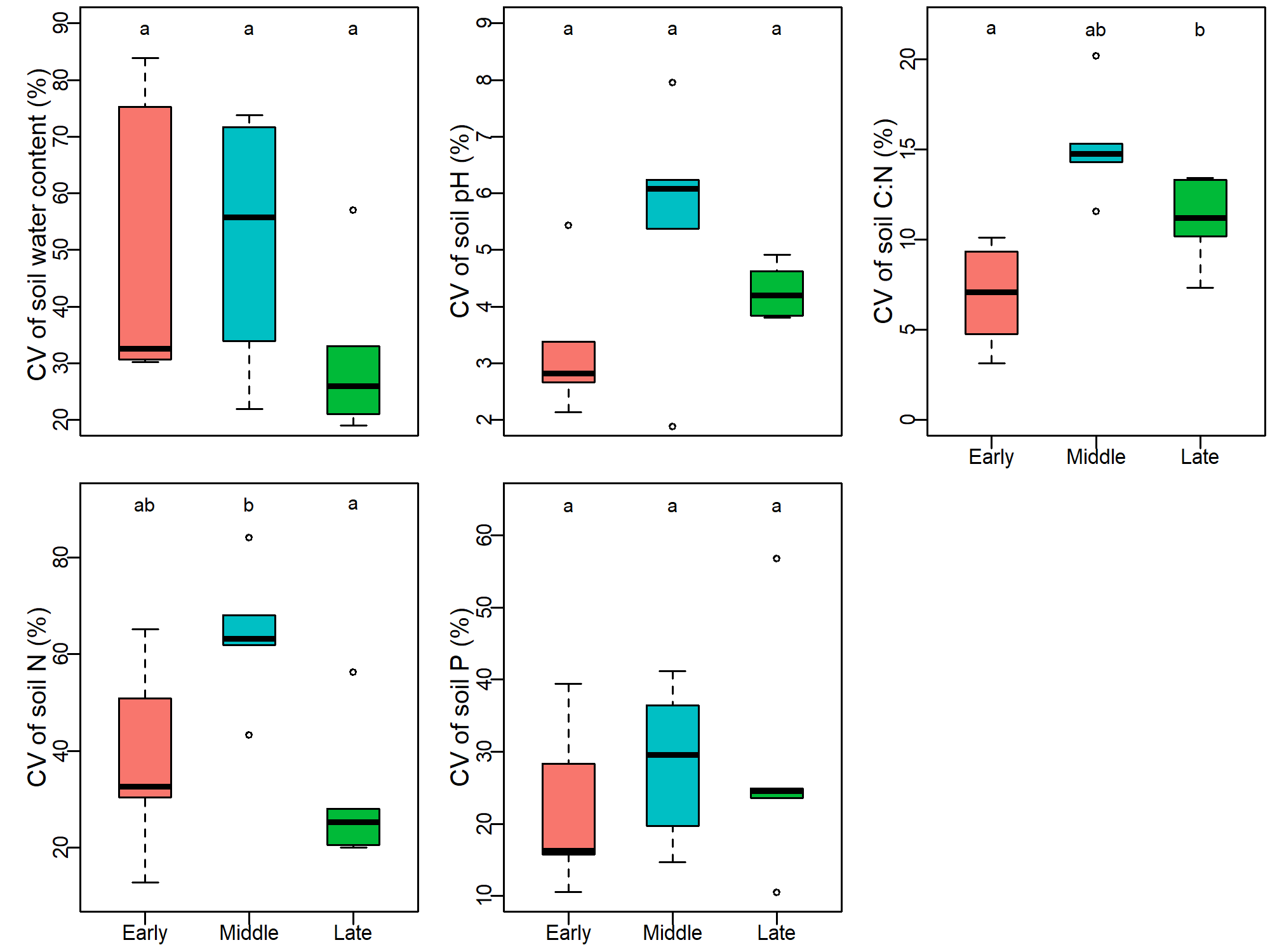


**Supplementary Figure S4.** Changes in coefficients of variation (CV) of soil properties during forest swamp succession. Different letters (a, b and c) indicate significant differences in CV of soil properties between successional stages (*p* < 0.05).

**1.2 Supplementary Tables**

**Supplementary Table S1.** Relationships between functional dispersion (FDis), species richness, soil factors, and soil heterogeneity.

|  | Leaf FDis | | | Root FDis | | |
| --- | --- | --- | --- | --- | --- | --- |
|  | *t_1,13_* | *R^2^* | *p* | *t_1,13_* | *R^2^* | *p* |
| Species richness | 0.97 | <0.01 | 0.352 | 1.71 | 0.12 | 0.112 |
| Soil factor |  |  |  |  |  |  |
| Soil pH | 4.27 | 0.55 | **<0.001** | 5.33 | 0.66 | **<0.001** |
| Soil C:N | -4.79 | 0.61 | **<0.001** | -5.52 | 0.68 | **<0.001** |
| Soil N | 3.76 | 0.48 | **0.002** | 2.54 | 0.28 | **0.025** |
| Soil P | 2.35 | 0.24 | **0.035** | 1.22 | 0.03 | 0.243 |
| Soil water content | 3.23 | 0.40 | **0.006** | 2.79 | 0.33 | **0.015** |
| Soil heterogeneity |  |  |  |  |  |  |
| CV of soil pH | 0.08 | <0.01 | 0.942 | -0.26 | <0.01 | 0.799 |
| CV of soil C:N | -0.60 | <0.01 | 0.556 | -0.94 | <0.01 | 0.367 |
| CV of soil N | 2.03 | 0.18 | 0.063 | 0.98 | <0.01 | 0.344 |
| CV of soil P | -0.24 | <0.01 | 0.816 | -0.67 | <0.01 | 0.516 |
| CV of soil water content | 1.88 | 0.15 | 0.083 | 1.23 | 0.04 | 0.239 |

Significant relationships are marked in bold (*p* < 0.05). CV, coefficient of variation.
